# Cell traction force in a confined microenvironment with double-sided micropost arrays†

**DOI:** 10.1039/c8ra10170a

**Published:** 2019-03-14

**Authors:** Jianan Hui, Stella W. Pang

**Affiliations:** Department of Electronic Engineering, City University of Hong Kong Hong Kong China pang@cityu.edu.hk; Center for Biosystems, Neuroscience, and Nanotechnology, City University of Hong Kong Hong Kong China

## Abstract

Three-dimensional (3D) cell migrations are regulated by force interactions between cells and a 3D extracellular matrix (ECM). Mapping the 3D traction force generated by cells on the surrounding ECM with controlled confinement and contact area will be useful in understanding cell migration. In this study, double-sided micropost arrays were fabricated. The cell traction force was mapped by microposts on the top and bottom of opposing surfaces with a controlled separating distance to create different confinements. The density of micropost arrays was modified to investigate the effect of cell contact area on 3D traction force development. Using MC3T3-E1 osteoblastic cells, the leading traction force was found to increase with additional contact surface on the top. Summing force vectors on both surfaces, a large force imbalance was found from the leading to trailing regions for fast migrating cells. With 10 μm separation and densely arranged microposts, the traction force on the top surface was the largest at 28.6 ± 2.5 nN with the highest migration speed of 0.61 ± 0.07 μm min^−1^. Decreasing the density of the top micropost arrays resulted in a reduced traction force on the top and lower migration speed. With 15 μm separation, the cell traction force on the top and migration speed further decreased simultaneously. These results revealed traction force development on 3D ECM with varied degrees of confinement and contact area, which is important in regulating 3D cell migration.

## Abstract 2

Double-sided micropost arrays to monitor three-dimensional cell traction force development over time on top and bottom surfaces with controlled confinement and contact area.

## Graphical abstract

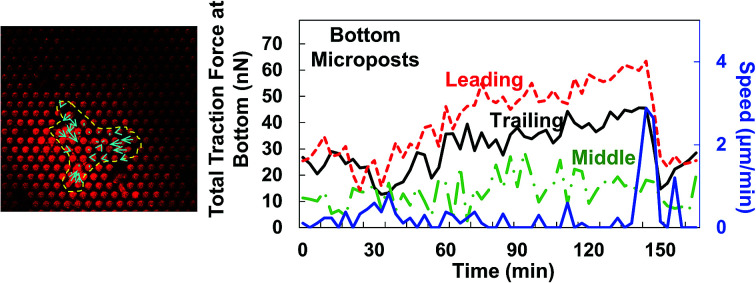

## Introduction

Cell migration studies inside three dimensional (3D) microenvironment are important because cells *in vivo* are surrounded by other cells and an extracellular matrix (ECM).^1–4^ The ECM *in vivo* is not a homogenous meshwork. It has a nonrandom form, such as “barrier-free” migration gaps in perivascular spaces and adipocytes^5^ and nanogrooved structures composed by periodical arrangement of collagen fibers in osteon units.^6,7^ These anisotropic 3D structures with different pore sizes and contacting surfaces can support and guide cell migration by regulating cell–ECM interactions through biochemical and physical means.^8–12^ Studies have shown that 3D cell migrations are largely affected by ECM-induced force transections that are either cell tractions generated at adhesive sites^13,14^ or intracellular pressure dependent on myosin-II mediated contractility and actin polymerization.^15,16^ Depending on the degrees of confinement and adhesions in 3D ECM, cell migration modes constantly switch among pseudopodial, lobopodial, bleb-based amoeboidal, or osmotic migrations,^17,18^ which are largely different from lamellipodial migration on two dimensional (2D) surfaces.^19,20^

Extensive studies have investigated the relationship between cell traction force and migration on 2D flat surfaces by measuring the traction force during the single and collective migration of epithelial cells,^21^ monitoring osteoblastic cell traction force development under topotaxis,^22^ and by mapping the traction force of dendritic cells during directional migration with chemotaxis.^23^ Cell migration on a 2D surface is determined by traction force evolvement, which is in steps of initial F-actin polymerization at cell leading region, protrusion of cell leading region with traction force at adhesive sites, and release and retraction of trailing edge.^24,25^ However, the biophysical mechanisms for 3D cell migration are different from those in 2D cell migration.^26,27^ For example, fibroblast cells in 3D fibrillar matrix were reported to have a slow retrograde flow, stabilized integrin-based adhesions, and enhanced directional migration that required myosin-II contractility, whereas cell migration speed increased on 2D surfaces when deprived of myosin-II contractility.^28,29^ Fibroblast cell migration underwent mesenchymal to amoeboid transition in platforms with 3 to 5 μm vertical confinement and low adhesive coatings.^15^ Previous studies included using micro-engineered platforms to analyze 3D cell migration,^30–32^ nucleus deformation,^33,34^ and cell–cell interactions.^35–37^ Despite biological models were further applied to explain 3D cell migration *in vivo*,^38,39^ few studies have investigated the physical force during cell migration under 3D contact and confinement.

Several methods were proposed to measure cell traction force in multiple dimensions. Fluorescent beads were embedded in hydrogel with cells seeded on top to measure the 2.5D traction force exerted by cells in both lateral and vertical directions against the ECM.^40^ Tractions generated from invading cells that were embedded in a 3D matrix were measured by tracking the 3D positions of fluorescent beads integrated in a collagen matrix^41,42^ or hydrogel.^43^ These methods measured cell tractions in 3D matrix are similar to native ECM *in vivo*. However, tuning porosity and stiffness in gel systems are limited. Hence, the quantitative study of cell traction force under controlled physical properties is difficult.^26^ Micropost arrays were integrated inside a longitudinal channel with a cross-sectional area varying from 10 × 4 to 50 × 4 μm^2^ to probe cell traction force under confinement with a defined stiffness.^44^ The cells were confined by a polydimethylsiloxane (PDMS) top lid and sidewalls, while traction force was measured by microposts at the bottom. As cells made contact with the PDMS top lid and sidewalls, traction force would disperse on the flat surfaces and could not be measured.

**Fig. 1:**
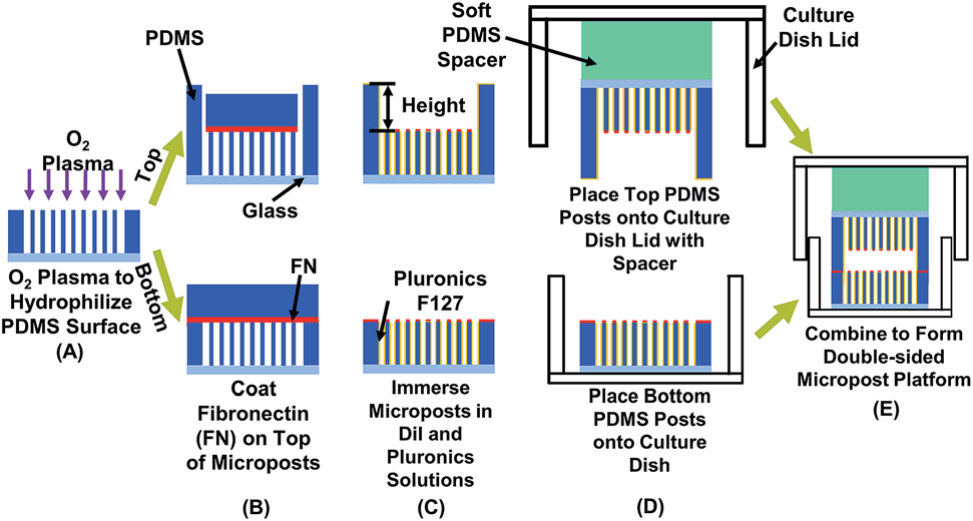
Schematic of fabrication technology for double-sided micropost platform to measure cell traction force in confined space. Effects of confinement and contact area were studied by modifying separation height and density of micropost arrays on top and bottom. (A) Using O_2_ plasma to hydrophilize polydimethylsiloxane (PDMS) microposts for coating fibronectin (FN). (B) Coating FN on top of microposts. (C) Blocking cell adhesion on sidewalls of microposts by covering sidewalls with Pluronic F-127 and labeling microposts with red stain DiI to enhance imaging contrast. (D) Placing top micropost platform on soft PDMS spacer and bonding to lid of 35 mm diameter (dia.) cell culture dish. Bottom micropost platform was placed on cell culture dish. (E) After seeding MC3T-E1 cells on bottom micropost platform, two parts were sealed to form double-sided micropost platform.

To investigate the relationships among cell traction force, physical confinement, and contact area, platforms integrated with opposing micropost sensing arrays at both top and bottom for measuring cellular force development on both contact surfaces were developed, as shown in Fig. 1. Osteoblastic cells were used as a cell model to study the effect of vertical confinement created by opposing surfaces on top and bottom, which are commonly seen *in vivo*. For example, bone cavity, brain vessels, fat tissues, and nerve tracks.^5,45^ Vertical confinements were formed by bordering sheet-like structures, which are generally the basement membrane, collagen bundles, and monolayers of lining cells. To study the 3D confinement effect, the separation distance between the top and bottom layer was changed and bottom from 10 to 15 μm. Cell migration was tracked, and the corresponding traction force development was monitored. The stiffness of the ECM was modulated by changing the dimensions of the micropost,^46,47^ considering that cells contacted only the top of the microposts. As osteoblastic cells are adherent cells sensitive to contacted surfaces, the density of micropost arrays on the top and bottom surfaces was modified to study how surface contacts affected traction force development during osteoblastic cell migration. Traction force development from the leading to trailing regions was compared during directional cell migration in real time. Traction force on both the top and bottom micropost layers was related to cell migration speed and range with different confinement and contact area. Therefore, the developed platforms could monitor the development of cell traction force during cell migration in 3D ECM with controlled confinement and surface contact. The results of this study could serve as a basis to decouple influential physical parameters in 3D ECM, model 3D cell migration *in vivo*, and design *in vitro* migration platforms to control cell migration in a confined 3D microenvironment. Also, the double-sided micropost platform could be used as an *in vivo* 3D microsystem to monitor cell behaviours, vitality,^48^ and responses after chemical treatment.^49^ The micropost arrays could be coated with various chemicals to assess the bio-compatibility of implants with different surface modifications.^50^

## Experimental

### Fabrication of PDMS micropost platforms on top and bottom

PDMS microposts were demolded from SU-8 master as previously described.^22^ To create a micropost layer on the bottom, a layer of SU-8 2010 (Microchem, MA, USA) was spin-coated on Si wafer and patterned by ultra-violet (UV) lithography to generate microposts with 3 μm in diameter (dia.). The distance between the micropost layers on the top and bottom was varied from 10 to 15 μm. The master for replicating the PDMS platform on top was created by spin-coating two layers of SU-8, exposing twice sequentially by UV, followed by a single development in accordance with previously described procedures.^51^ All fabricated SU-8 masters were treated with trichloro(1*H*,1*H*,2*H*,2*H*-perfluorooctyl)silane (FOTS) (Sigma-Aldrich, WI, USA) to form an anti-sticking layer. A PDMS prepolymer (base monomer : curing agent weight ratio = 10 : 1, Sylgard 184, Dow Corning, MI, USA) was poured on SU-8 masters, cured in a 110 °C convection oven for 6 h, and demoled onto a cover glass after being fully cross-linked. PDMS microposts were kept in upright position by ultra-sonicating in absolute ethanol (≥99.8%, Sigma-Aldrich, WI, USA) and dried with a critical point dryer (EM CPD300, Leica, Hesse, Germany).

The patterned PDMS platform was treated with an O_2_ plasma for successful coating of both fibronectin (FN) and Pluronic F-127, as shown in Fig. 1A. A microwave plasma ashing system (GIGAbatch 310 M, PVA TePla, Wettenberg, Germany) was used with 135 sccm O_2_, 15 sccm N_2_, 150 mTorr, and 30 W rf power within a Faraday cage for 15 s. The contact angle of deionized (DI) water was measured to be 93.1 ± 2.0 after plasma treatment, as shown in Fig. S1.† A PDMS stamp was incubated with FN (50 μg ml^−1^ in DI water, Sigma-Aldrich, MO, USA) for 6 h, rinsed in DI water, and then blow dried with N_2_. The FN coated PDMS stamp contacted and transferred the FN to the top of microposts on both the top and bottom layers, as shown in Fig. 1B. Absolute ethanol was added for easy separation of the PDMS stamp from the microposts and for disinfection of the platforms. The platforms were immersed in 70% ethanol and then rinsed twice with phosphate-buffered saline (PBS). To enhance the image contrast for analyzing micropost displacement, the micropost arrays were labeled with DiI (5 μg ml^−1^ in distilled water, 1,10-dioleyl-3,3,30,30-tetramethylindocarbocyanine methanesulfonate, Invitrogen, CA, USA) stain by submerging the platforms for 90 min in room temperature. After rinsing in PBS three times, 0.2% Pluronic F-127 (Sigma-Aldrich, WI, USA) was added and incubated for 30–60 min to allow the coating of Pluronics on the sidewalls of microposts, as shown in Fig. 1C. The coating of FN on top and Pluronics on sidewalls controlled cell adhesions only on top of microposts.^52^

The top PDMS platform was bonded to a PDMS spacer (base monomer : curing agent weight ratio = 35 : 1) and lid of cell culture dish by an O_2_ plasma treatment (GIGAbatch 310 M, 135 sccm O_2_, 15 sccm N_2_, 150 mTorr, and 50 W rf power for 15 s). The soft PDMS spacer ensured uniform contact between the top and bottom PDMS platforms.^53^ The bottom PDMS platform was glued onto a culture dish substrate with PDMS prepolymer, as shown in Fig. 1D. After placing the PDMS platforms on the lid and bottom of the cell culture dish, they were immersed in PBS solution for cell migration study.

### Cell culture and seeding on PDMS platforms

MC3T3-E1 osteoblastic cells were obtained from American Type Culture Collection (ATCC numbers CRL-2594) and maintained in high glucose Dulbecco's modified eagle medium (DMEM, Invitrogen, CA, USA), supplemented with 10% fetal bovine serum (FBS, Gibco, MD, USA), antibiotic-antimycotic (100 units per ml of penicillin, 100 mg ml^−1^ of streptomycin, and 0.25 mg ml^−1^ of amphotericin B, Gibco, MD, USA), and 2 mM alanyl-l-glutamine (Gibco, MD, USA). Cells were incubated at 37 °C and 5% CO_2_ with culture medium changed every 3 days. Bone formation includes states of activation and termination of bone cells. From stages of activation to termination, the osteoblastic cells become mature and the cellular behaviour changes dramatically. For example, mature osteoblast would become immobilized and differentiate into osteocyte.^54^ Hence, osteoblastic cells were passed every 5 days to keep below full confluence at all times. The MC3T3 cell passage was controlled in the range of 3 to 20 and all cells were constantly moving during time-lapse imaging. The designed platforms could be used for cells in different states.

Before seeding on the micropost platform, the MC3T3-E1 cells were trypsinized (0.05% w/v trypsin in EDTA) for 8 min for detachment. Cell density of 1 × 10^4^ cells per ml was seeded onto the bottom micropost platform and maintained at 37 °C and 5% CO_2_ for 15 h to allow complete cell adhesion. The top microposts were also submerged in DMEM and incubated at 37 °C and 5% CO_2_ to ensure a similar condition as microposts at bottom. The DMEM in bottom micropost platform was then replaced by a CO_2_ independent medium (Invitrogen 18045-088, CA, USA), 10% FBS, antibiotic–antimycotic, and supplemented with 2 mM alanyl-l-glutamine (Gibco, MD, USA). The top micropost platform was aligned with the bottom platform and fixed in place with tape, as shown in Fig. 1E.

### Observation of cell migration under time-lapse confocal microscope

A laser scanning confocal microscope (TCS SP5, Leica, Hesse, Germany) was used to take time-lapse images at a time interval of 3 min for 6 h. Focus along *z* axis was changed from bottom to top PDMS platforms to check the separation distance between microposts on two sides. The stage movement along *xy*-plane was fixed for stable focusing. To measure the cell traction force on the top and bottom micropost layers during cell migration, a 20× oil immersion objective lens was used with a 60 μm pinhole. Vertical *z*-plane was focused on the top surface of the microposts during timelapse imaging. To acquire the bending of the PDMS post, the top position of the micropost was compared with its original position. For high contrast imaging, the DiI stain was excited at 543 nm and detected at 570 nm. Bright field images were also taken to show cell positions for analysis of cell migration. Before and during imaging of cells in the double-sided micropost platforms, the MC3T3-E1 osteoblastic cells were healthy and moved on the top surface of microposts freely and normally. Even after seeding and observing under the confocal microscope for over 15 h, cells were lively, indicating the 3D platforms were safe microenvironment for cells.

### Cell morphology by scanning electron microscopy

To observe cell morphology, cells were fixed on PDMS platforms and observed under a scanning electron microscope (SEM). After confocal imaging for 6 h, the cell culture dish was opened and top micropost layer was separated from the bottom layer. Cells were rinsed with 37 °C PBS and fixed in 4% (w/v) 37 °C paraformaldehyde (PFA, Sigma-Aldrich, WI, USA) in PBS for 15 min at room temperature. After washing the excessive PFA thoroughly with PBS three times, the PBS in the PDMS platforms was replaced by an ascending concentration of series of ethanol (30%, 50%, 70%, 80%, 90%, 95%, and 100%). To reduce the artifacts created by surface tension, the cells were supercritically dried using critical point dryer (EM CPD3000, Leica, Hesse, Germany). A thin layer of gold was coated on the platforms to avoid charging. A field emission SEM (SU5000 FE-SEM, Hitachi, Tokyo, Japan) was used to image the coated samples.

### Mechanical properties of microposts

The dimensions of the fabricated microposts were determined using SEM. The microposts arrays were in hexagonal arrangement with the dia. of 3 μm and height of 13.4 μm, as shown in Fig. S2.† The spacing from edge to edge between two adjacent microposts at bottom layer was 3 μm. The spacing for microposts on the top layer was varied from 3 to 5 μm to study the effect of cell contact area during confined migration. Curing for 6 ± 0.5 h in a 110 °C oven was used to fully cross link the 10 : 1 PDMS. The corresponding PDMS Young's modulus was 2.5 MPa. When cells only contacted the top of microposts, each post was treated as a cylindrical cantilever beam with one end fixed to the substrate and the other end loaded with lateral cell traction force. The mechanical property of the PDMS posts depended on the micropost dimensions and Young's modulus of PDMS.^46^ The rigidity of a single PDMS post was characterized using the finite element method and assuming the PDMS Young's modulus to be 2.5 MPa. The calculated spring constant, *k*, was 8.84 nN μm^−1^.^22^

In order to measure the distance between the microposts on top and bottom layers, the focus along the vertical direction was changed. To investigate the effect of contact area on cell migration with confinement, cells were tracked inside confined space with flat surfaces or microposts. The density of microposts on the top layer was changed, with 3 to 5 μm spacing between microposts, to vary the cellular contact area.

### Data analysis

Cell migration trajectories and speed were tracked by Manual Tracking plugin of NIH ImageJ software (version 1.48v). To detect the displacement of the microposts, a MATLAB (R2007b, The MathWorks, MA, USA) graphical user interface (GUI) is used to process the images.^22,46^ Fluorescent images focused at the base and top of the posts were loaded into the GUI to calculate the lateral displacement, Δ*x*, of the top of microposts. The cell traction force was calculated by multiplying nominal spring constant with micropost displacement, *F*_traction_ = *k* × Δ*x*. All quantitative results were presented with mean ± standard error (SE). Statistical significance was tested by employing the Student's *t*-test and null hypothesis was rejected when *p* < 0.05. Three individual experiments were taken for the analysis of cell tracking and traction force.

## Results and discussion

### Cell migration trajectories and speed under vertical confinement

Vertical confinement is one of the basic models where cells migrate in between two opposite layers *in vivo*, such as the space between two peritoneal layers covering the inner organs, bone cavities covered by monolayers of osteoblastic cells, and perivascular space between the pia mater and elastic membrane covering the smooth muscle cells inside brain vessels.^5,55^ Cell migration was found to be regulated by cell contact with 3D ECM and cell traction force on adhesive sites.^13^ To understand cell migration during cellular contacts with a 3D ECM, cell migration was tracked with different separation distances.

**Fig. 2:**
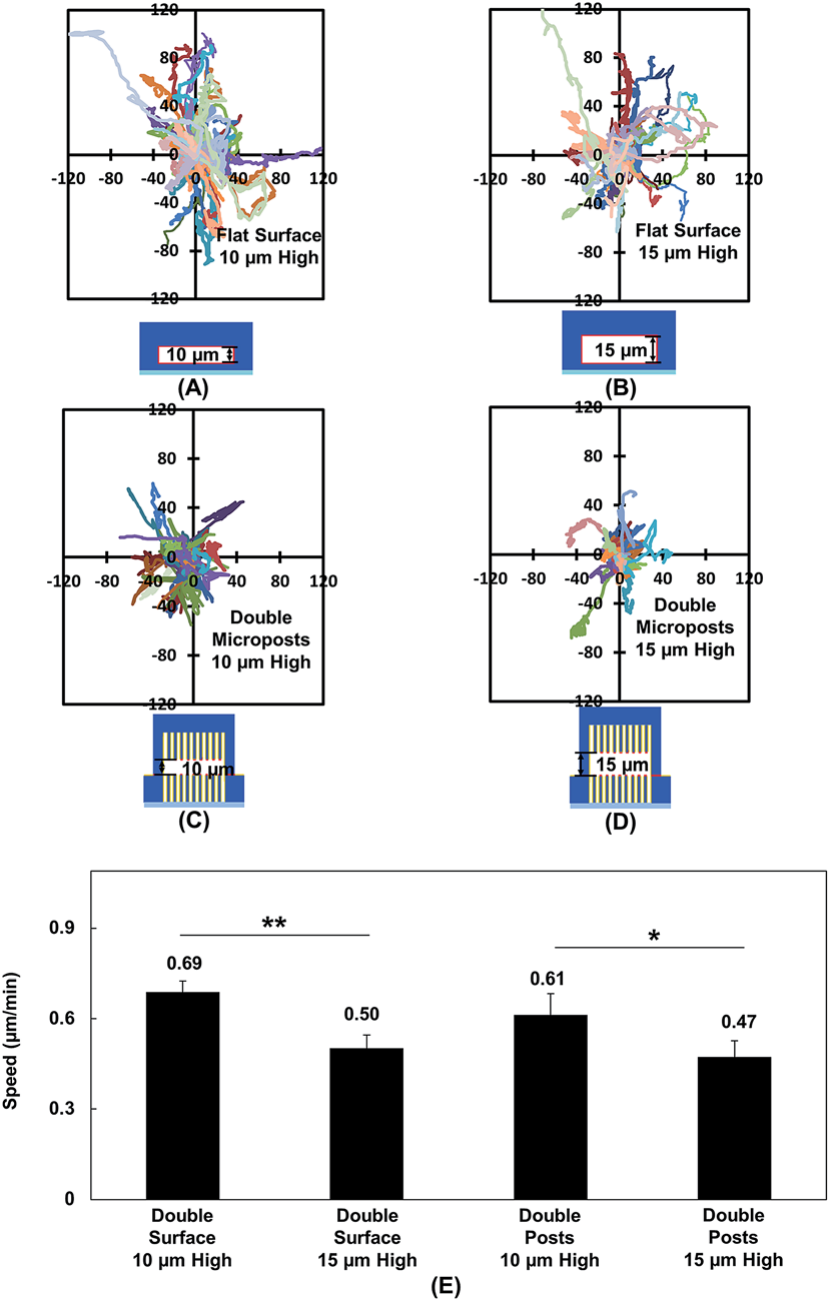
(A and B) Cell migration trajectories in confined platforms with flat surfaces on both top and bottom with height of 10 (*N* = 67) and 15 μm (*N* = 68). (C and D) Trajectories of cell migration inside double-sided micropost arrays with separation height of 10 (*N* = 36) and 15 μm (*N* = 25). (E) Cell migration speed inside confined platforms with flat surfaces or micropost arrays on top and bottom. Micropost arrays on both sides of platform were 3 μm in dia. and 3 μm spacing. Statistical significance was calculated using Student's *t*-test (*p < 0.05 and **p < 0.01).

Fig. 2A–D show the MC3T3-E1 cell migration trajectories inside confined spaces with opposing surfaces separated by 10 and 15 μm. When the cells migrated in between flat surfaces with a separation of 10 μm (Fig. 2A), they moved with a larger range compared with the cell migration in between surfaces with 15 μm separation (Fig. 2B). A similar cellular behavior was observed when cell migration in between double-sided microposts with 10 and 15 μm separation was compared. Fig. 2C suggests that more cells showed a larger range inside the 10 μm separation between the double-sided microposts compared to the 15 μm separation, as shown in Fig. 2D. Fig. 2E shows that the average cell migration speed of 0.69 ± 0.03 μm min^−1^ in between double surfaces with 10 μm separation was higher than the cell migration speed of 0.50 ± 0.04 μm min^−1^ in between double surfaces with 15 μm separation. For the double sided post arrays, the cells migrated faster at 0.61 ± 0.07 μm min^−1^ when separated by 10 μm compared to 0.47 ± 0.05 μm min^−1^ when separated by 15 μm. Cells showed a larger migration range and faster speed in between 10 μm separation, which was similar to previous reports that the migration of fibroblast cells in pore size comparable to cell nucleus is faster.^56^

### Cell traction force development and cell spreading in between double-sided microposts with 10 or 15 μm separation

**Fig. 3:**
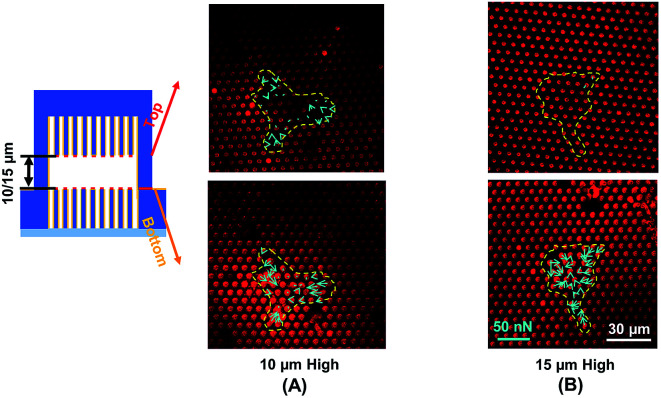
Traction force measured using double-sided micropost arrays with (A) 10 μm and (B) 15 μm separation. Focal planes along *z*-axis were on top of microposts. Microposts were 3 μm in dia. with 3 μm spacing.

Traction force developed on both the top and bottom micropost layers with 10 μm separation is shown in Fig. 3A. On the top side of the micropost layer, traction force developed only at the edges of the cell. On the bottom micropost layer, larger traction force developed around the lamellipodia. The force direction on both sides was similar and centripetal. When the separation was increased to 15 μm, larger traction force was developed at the bottom layer compared to the top layer, as shown in Fig. 3B. The cells generated a small traction force on top micropost layer, whereas the traction force at the bottom layer was larger and comparable with the 10 μm separation.

**Fig. 4:**
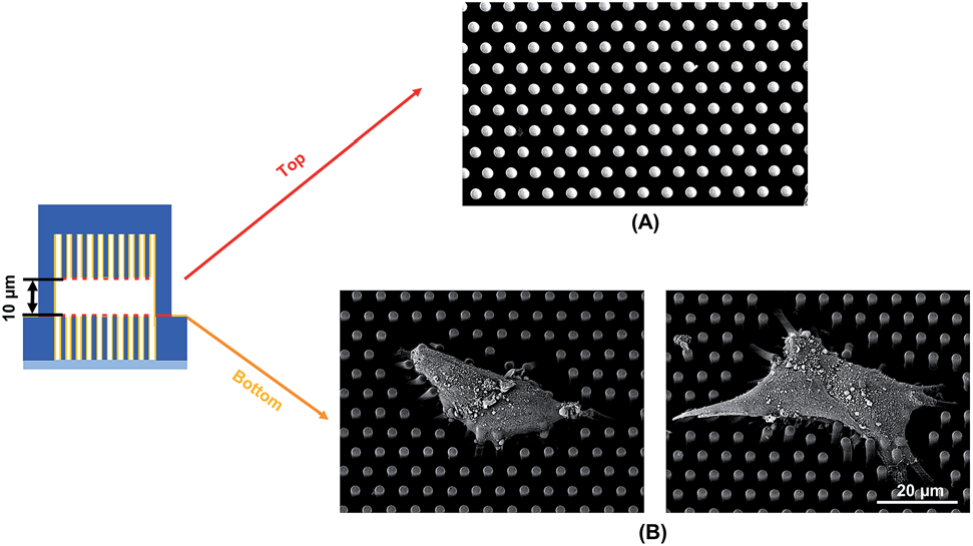
Scanning electron micrographs showing cell spreading and adhesion on (A) top and (B) bottom microposts with 10 μm separation. Microposts were 3 μm in dia. with 3 μm spacing.

To observe the cell spreading on both layers, the sealed structures with 10 μm separation was imaged using a SEM. Fig. 4 shows the cell morphology on both top and bottom layers. No cell remained on the top layer, as shown in Fig. 4A. Cells were attached to the bottom layer and generated large traction force around cell edges on the top of the microposts, as shown in Fig. 4B. Some cells detached after the top and bottom layers were separated, and the cells with strong adhesions at the bottom layer remained. Although cells could generate traction force on both the top and bottom layers with 10 μm separation, the adhesions were stronger at the bottom layer with higher traction force. The cells barely generated adhesion on the top layer with 15 μm separation, and traction force mostly formed at the bottom micropost layer.

### Monitoring cell traction force on top and bottom micropost arrays during forward cell migration with 10 μm separation

**Fig. 5:**
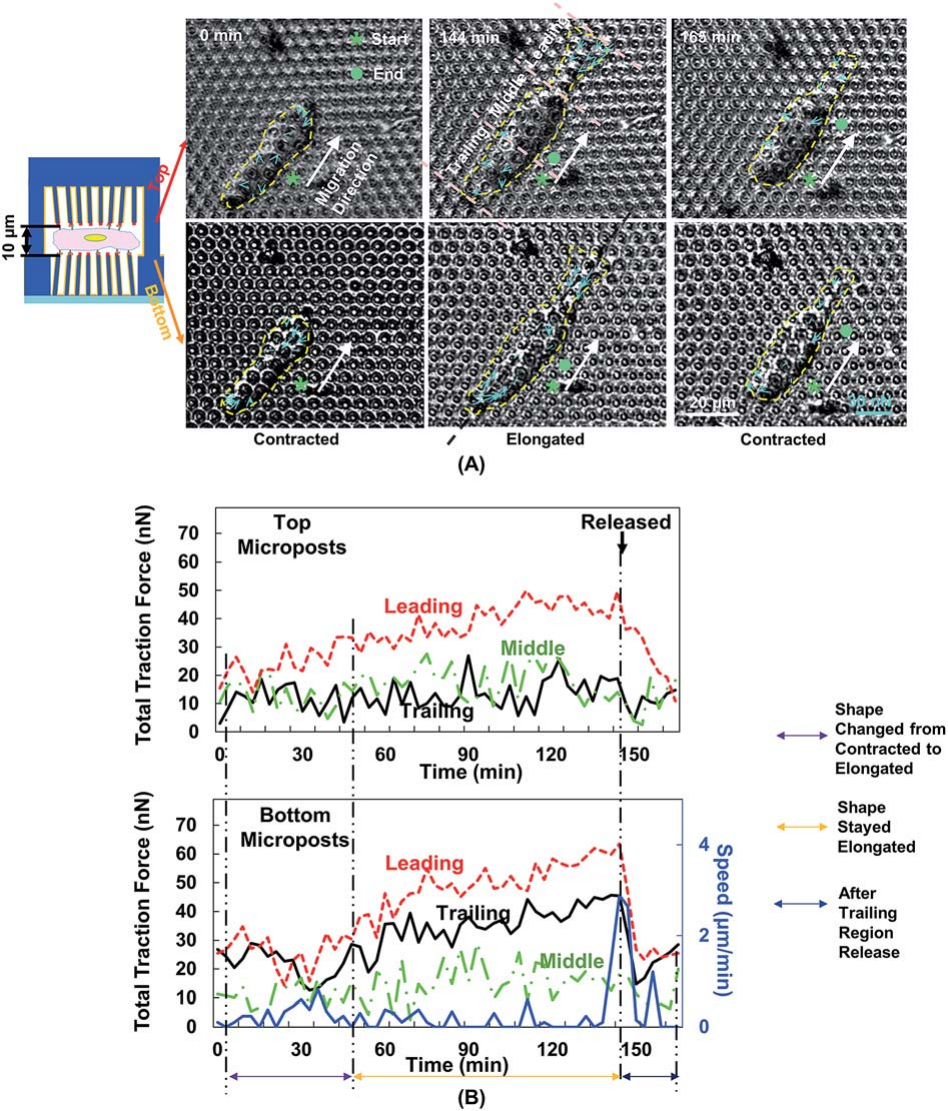
Force mappings during cell migration in double-sided micropost platform with 10 μm separation. (A) Cell started migration in contracted shape. When leading region protruded from 0 to 144 min, cell became elongated and traction force increased. Cell released its trailing region and became contracted again from 144 to 165 min, after which it started second migration cycle. Cellular contour is indicated by yellow dashed line. Starting and ending positions are indicated by asterisk and dot, respectively. Migration direction is described as shift of centroid of cell from beginning to end during single migration cycle, which is labelled using white arrow. Cell traction force is indicated by blue arrows. (B) Cell traction force and speed were analysed on both top and bottom micropost arrays during forward cell migration. Total traction force was analysed by adding force vectors in leading, middle, and trailing regions. Black arrow indicates moment when trailing region was released, starting at 144 min. Microposts were 3 μm in dia. with 3 μm spacing.

Fig. 5A shows the changes in cell morphology and traction force on the top and bottom micropost layers with 10 μm separation. At 0 min when MC3T3-E1 cells started forward migration, the cells showed a contracted shape and the traction force was small across the cellular body on both sides. Then, the cells started to protrude the leading edge and became elongated from 0 to 144 min. At the bottom micropost layer, traction force in both the leading and trailing regions increased and pointed towards the cell centre. On the top side, the traction force in the leading region increased and pointed toward the cell centre, whereas the traction force in the trailing region was randomly distributed. From 144 to 165 min, the cell released its trailing region and its shape became contracted again. The traction force in both the leading and trailing regions decreased, and the force direction became less centripetal. The cyclic changes of the cell shape and traction force on the top and bottom layers continued as the cell kept migrating forward.

We have previously reported that the dynamic changes of the resultant traction force in the leading, middle, and trailing regions could regulate cell migration direction.^22^ In the present study, the cell was similarly divided into three regions along its long axis, and the resultant force was obtained by the addition of force vectors in each region. Fig. 5B shows the development of cell migration speed and total traction force in the leading, middle, and trailing regions. When the leading region was protruded from 0 to 48 min, the traction force gradually increased in both leading and trailing regions. As the cell became elongated, its migration speed decreased, and no obvious locomotion of the cell centroid was observed. The traction force in the leading and trailing regions continued to build up, and the traction force in the leading region was larger than that in the trailing region. As the trailing region was released from 144 to 165 min, traction force at both leading and trailing regions dropped and the speed reached a maximum level.

The polymerization of F-actin in the leading region and the contractility produced by myosin-II were shown to pull the cells to migrate on 2D surface.^25^ The cells showed a similar traction force development on the bottom layer during cell migration in double-sided micropost platforms. However, the traction force on the top micropost layer remained small in both trailing and middle regions. In the leading region of the cell, the traction force development was similar to the bottom micropost layer. An increment of traction force was observed as the cell shape changed from contraction to elongation. The traction force rapidly dropped as the cell lost adhesion in the trailing region. The amount of traction force was smaller than the force generated at the bottom layer despite similar force development trend. The leading region of the cell protruded and generated adhesion with the top micropost layer. The traction force in the middle and trailing regions did not change with time. Therefore, the cell migration was mainly controlled by the contact with top micropost layer in the leading region.

### Comparison of cell traction force from leading to trailing regions during forward cell migration

**Fig. 6:**
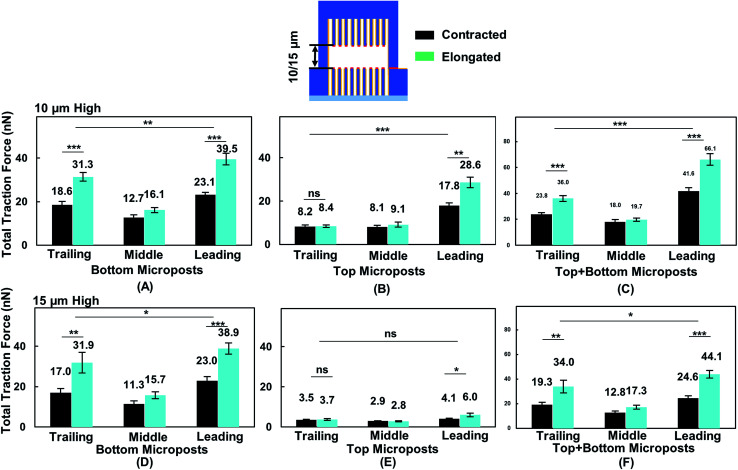
Traction force on top and bottom of platform in leading, middle, and trailing regions while cell shape changed from contraction to elongation. With 10 μm separation (*N* = 12), cell traction force was measured on (A) bottom, (B) top, and (C) both sides of microposts. For 15 μm separation (*N* = 11), cell traction force was measured on (D) bottom, (E) top, and (F) both sides of microposts. Force was analysed by adding force vectors. Microposts were 3 μm in dia. with 3 μm spacing. Statistical significance was calculated using Student's *t*-test (***p < 0.001).

To correlate cell migration with traction force development, cell traction force on the top and bottom micropost layers with 10 and 15 μm separation was measured. The cell traction force at the bottom micropost layer with 10 μm separation was small in both the leading (23.1 ± 1.2 nN) and trailing (18.6 ± 1.6 nN) regions when the cell was contracted, as shown in Fig. 6A. The cell traction force increased in the leading (39.5 ± 2.7 nN) and trailing (31.3 ± 2.0 nN) regions as the cell elongated from contraction. On the top micropost layer, the traction force in the leading region also increased from 17.8 ± 1.4 to 28.6 ± 2.5 nN when the cell shape changed from contraction to elongation, as shown in Fig. 6B. However, no change in the traction force in the trailing region was observed when the cell contracted (8.2 ± 0.8 nN) or elongated (8.4 ± 0.7 nN). The cell traction force in the leading region was always the largest among the three regions on both the top and bottom micropost layers. The traction force in the trailing region was larger than that in the middle region at the bottom layer while there was no difference for the top layer. Fig. 6C shows that the traction force difference between the leading and trailing regions further increased when both the traction force generated on the top and bottom layers were included. When the cell contracted, it generated a traction force of 41.6 ± 2.6 nN in the leading region and 23.8 ± 1.3 nN in the trailing region. The total traction force on the top and bottom layers in the leading region increased to 66.1 ± 4.5 nN and was significantly larger (*p* = 0.000012) than 36.0 ± 2.3 nN in the trailing region as the cell became elongated.

During forward cell migration in double-sided microposts with 15 μm separation, the relationship of traction force for the bottom micropost layer in the leading, middle, and trailing regions was similar to the 10 μm separation, as shown in Fig. 6D. On the top micropost layer, the total traction force was smaller than that with 10 μm separation, as shown in Fig. 6E. As the cell shape changed from contraction to elongation, the traction force in the leading region developed on top changed from 4.1 ± 0.3 to 6.0 ± 0.9 nN and it remained unchanged in the trailing region. No significant difference was found between the leading and trailing regions on the top side. Fig. 6F shows the total traction force on the top and bottom layer was 24.6 ± 2.0 nN in the leading region and 19.3 ± 1.8 nN in the trailing region when the cell contracted. As the cell became elongated, the traction force was 44.1 ± 3.1 nN in the leading region and 34.0 ± 5.1 nN in the trailing region, and the difference was not as large (*p* = 0.017) as that for the 10 μm separation. Therefore, the cells could protrude its leading region and generate larger traction force on the top micropost layer than the middle and trailing regions during forward migration in between double-sided microposts with 10 μm separation. Adding the traction force on both sides of the microposts, the difference between the leading and trailing regions was larger with 10 μm separation.

### Cell migration and traction force development during forward migration in double-sided micropost arrays with 10 μm separation

MC3T3-E1 cells showed different migration behaviors when they moved in between double-sided microposts in comparison to flat surfaces with the same height. To further validate the effect of cellular contact and correlated traction force development on cell migration, the density of microposts between the top and bottom layers was varied. The micropost arrays on the bottom side were 3 μm in dia. and 3 μm in spacing. The spacing was changed from 3 to 5 μm, and the dia. remained at 3 μm for the top layer. The separating distance between the top microposts with 5 μm spacing and bottom with 3 μm spacing was kept at 10 μm to enable cell contact with both surfaces. The force mapping of a typical cell spread in between double-sided micropost arrays with different densities is shown in Fig. S3.† With 10 μm separation, the cell contacted and developed traction force on both top and bottom micropost layers. On the top micropost layer with 5 μm spacing, a traction force was observed around cell edges.

**Fig. 7:**
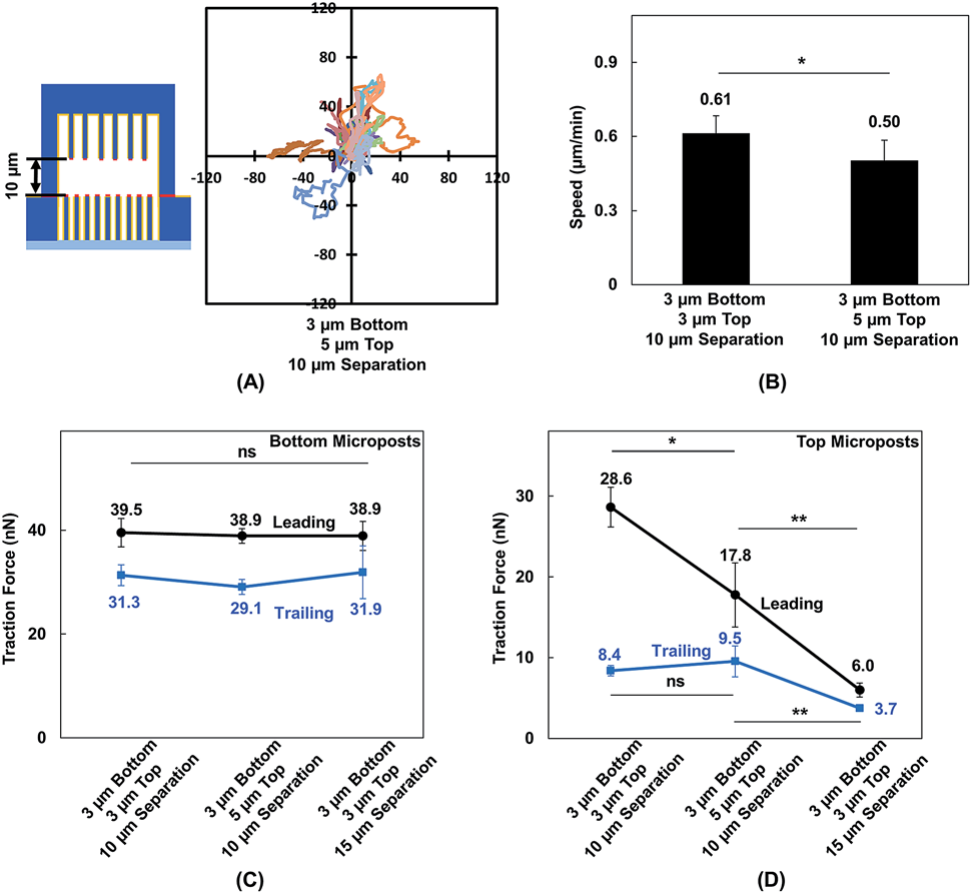
(A) Cell migration trajectories (*N* = 20) in double-sided micropost platform with 10 μm separation. Micropost array at bottom was 3 μm in dia. with 3 μm spacing. Spacing was 5 μm for top microposts. (B) Cell migration speed in platforms with 10 μm separation between top and bottom micropost arrays with micropost spacing of 3 (*N* = 36) and 5 μm (*N* = 20) on top side. Comparison of traction force when cells became elongated on platforms with 3 μm spacing and 10 μm separation, 5 μm spacing and 10 μm separation, and 3 μm spacing and 15 μm separation. Traction force in leading and trailing regions generated on (C) bottom microposts and D: top microposts. All microposts were 3 μm in dia.

Cell migration trajectories were tracked in double-sided micropost arrays with 5 μm spacing and 10 μm separation, as shown in Fig. 7A. The migration range was smaller compared with the cell migration in double-sided microposts with 3 μm spacing and 10 μm separation, as shown in Fig. 2C. The average speed was also compared among the microposts of varied densities on top, as shown in Fig. 7 B. The cell migration speed was 0.50 ± 0.08 μm min^−1^ in between double-sided microposts with 5 μm spacing and 10 μm separation. This speed was slower compared with the 0.61 ± 0.07 μm min^−1^ for cell migration in between double-microposts with 3 μm spacing and 10 μm separation.

To correlate the cell migration speed with traction force development, the cell traction force as the cells became elongated during migration in between double-sided micropost arrays with 3 μm spacing on top with 10 μm separation, 5 μm spacing on top with 10 μm separation, and 3 μm spacing on top with 15 μm separation was compared. For the bottom micropost layer, the cell traction force was similar in the leading and trailing regions for all platforms, as shown in Fig. 7C. Traction force changed on the top micropost layers during forward migration, as shown in Fig. 7D. The highest traction force of 28.6 ± 2.5 nN was obtained in the leading region during migration in between double-sided micropost arrays with 3 μm spacing on top and 10 μm separation. With 10 μm separation and spacing increased to 5 μm on top, the cell traction force in the leading region decreased to 17.8 ± 4.0 nN. When the separating distance was increased from 10 to 15 μm, the cell generated the smallest traction force of 6.0 ± 0.9 nN in the leading region and had the least interactions with the micropost arrays on top. In the trailing region of the cell migrating in double-sided microposts with 10 μm separation, no difference was observed in terms of traction force generated on the top surface with different densities. Cells could still make contact in the trailing region, but the traction force was small and direction was not centripetal, as shown in Fig. 5. When separation was increased to 15 μm, cells barely made contact with the microposts on top and hence the traction force in the trailing region decreased.

MC3T3-E1 cells could generate stable adhesions on the top of micropost while they made contacts. When the separating distance was reduced, the cells generated higher traction force in the leading region.^57,58^ As the micropost density on the top layer increased, the total traction force in the leading region also increased. The relationship between the density of microposts and traction force was also studied on micropost platforms with 3 and 5 μm spacing, as shown in Fig. S4.† As cells became elongated, the traction force in the leading and trailing regions were 38.1 ± 3.4 and 29.4 ± 3.5 nN, respectively, on densely arranged micropost arrays with 3 μm spacing. The total force reduced to 33.3 ± 2.2 nN in the leading region and 26.1 ± 2.0 nN in the trailing region when the spacing between microposts increased to 5 μm. Adhesion-based cell migration in 3D ECM depends on the pulling force generated in the leading region of the cell and the rate for the nucleus to squeeze through confined spaces in the trailing region.^56,57^ As 10 and 15 μm layer separations approach the size of the nucleus, the decreased separating distance from 15 to 10 μm and increased micropost density could increase total traction force generated in the leading region and help to pull the cell migrate forward.

## Conclusions

In this study, MC3T3-E1 osteoblastic cell migration was tracked in between opposing flat surfaces and double-sided micropost arrays with different separating distances. Cells with 10 μm separation between flat surfaces migrated in a larger range with a higher speed of 0.69 ± 0.03 μm min^−1^ compared with cells with 15 μm separation between flat surfaces (0.50 ± 0.04 μm min^−1^). Similarly, the cell migration speed in between double-sided micropost arrays with 10 μm separation was 0.61 ± 0.07 μm min^−1^ and was higher than that in between 15 μm-separated micropost arrays with a speed of 0.47 ± 0.05 μm min^−1^.

To correlate cell traction force development during cell migration under confinement, the cell traction force on the top and bottom micropost layers was measured during forward cell migration in between opposing surfaces with the separating distance changed from 10 to 15 μm. Similar to 2D lamellipodial migration in the forward direction, the cyclic development of cell traction force in the leading, middle, and trailing regions was observed at the bottom micropost layer. Cell adhesion and traction force on the top surface became weaker when the separation was 15 μm. By decreasing the separation distance from 15 to 10 μm, the traction force on the top micropost layer showed correlation with cell shape and speed during forward cell migration. As a cell protruded its leading lamella and became elongated, the traction force in the leading region increased and showed a larger difference compared with that in the trailing region. The total traction force in the leading region generated on both surfaces with 10 and 15 μm separation was 66.1 ± 4.5 and 44.1 ± 3.1 nN, respectively. In the trailing region, the total traction force with 10 and 15 μm separation were 36.0 ± 2.3 and 34.0 ± 5.1 nN, respectively. A larger significant difference between the leading and trailing regions was observed with 10 μm separation (*p* = 0.000012) than with 15 μm separation (*p* = 0.017).

The contact effect in 3D ECM was also investigated by modifying the micropost density on top. The spacing between microposts on top was increased from 3 to 5 μm, whereas the spacing between microposts at the bottom remained at 3 μm. As the spacing on the top microposts increased from 3 to 5 μm with 10 μm separation, the migration speed decreased from 0.61 ± 0.07 to 0.50 ± 0.08 μm min^−1^, and the leading region traction force decreased from 28.6 ± 2.5 to 17.8 ± 4.0 nN on top micropost layer. When the separation distance was increased from 10 to 15 μm, the cell migration speed and leading traction force on top further decreased.

Therefore, the traction force generated in the leading region was primarily determined by the separating distance and was also affected by the density of microposts. Higher traction force produced at the leading region of cells during migration in 3D ECM created a larger force imbalance from the leading to trailing regions, which resulted in higher cell migration speed. To our best knowledge, this is the first study to map the dynamic development of cell traction force on top and bottom surfaces cell contacted in a 3D microenvironment. These results will provide better understanding of adherent cell migration in 3D ECM *in vivo*. Other cellular types with different migration modes and cellular states, such as tumour cells with the ability to switch between amoeboid-mesenchymal movements when activated, can be seeded on similar platforms to investigate the physical mechanisms of cell migration. Hence, these double-sided micropost sensing arrays provided broad applications and the results of 3D cell traction force are useful for designing platforms to control cell migration.

## Conflicts of interest

There are no conflicts to declare.

## Supplementary Material

RA-009-C8RA10170A-s001
