# Supplementary material for: Cell traction force in a confined microenvironment with double-sided micropost arrays
Source: RSC Adv. 2019 Mar 14;9(15):8575–84. doi: 10.1039/c8ra10170a (PMC9061871; doi:10.1039/c8ra10170a)
Supplement: RA-009-C8RA10170A-s001 [file RA-009-C8RA10170A-s001.pdf]

Electronic Supplementary Material (ESI) for RSC Advances.
This journal is © The Royal Society of Chemistry 2019

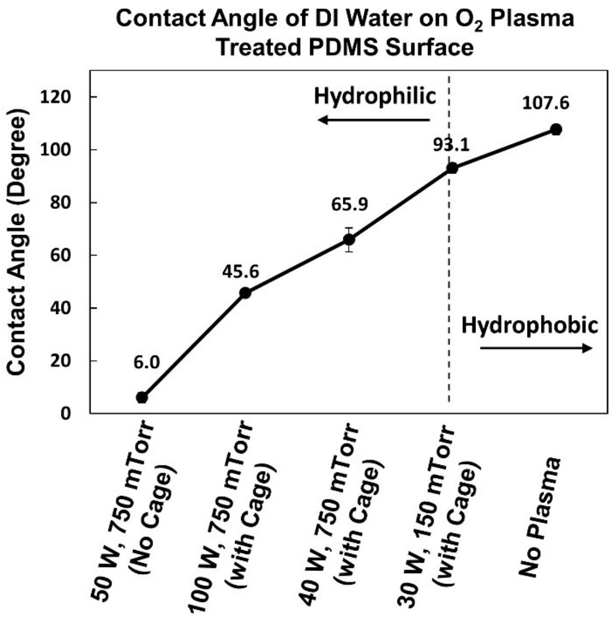

Figure S1

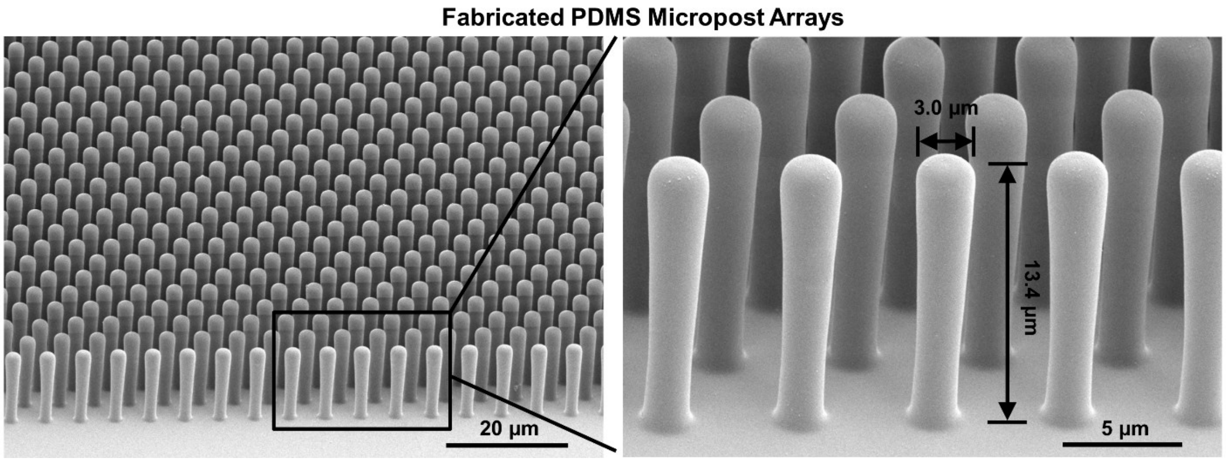

Figure S2

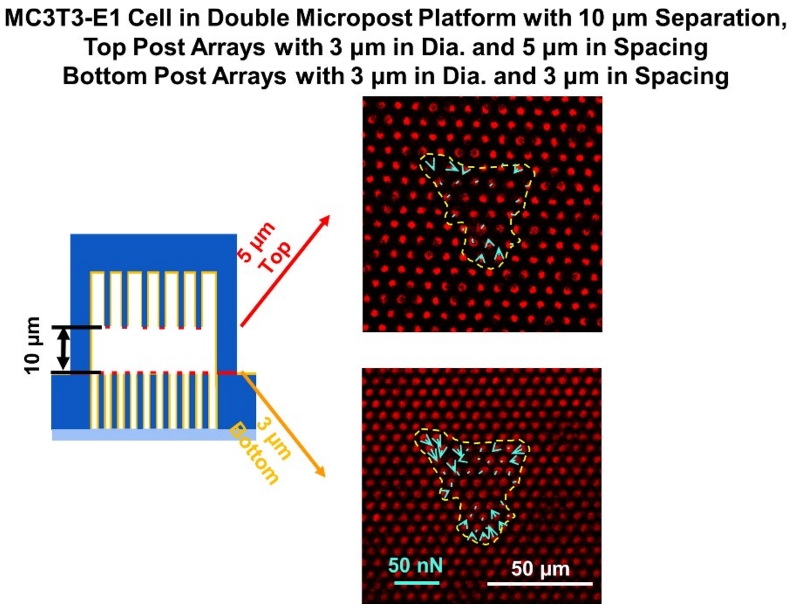

Figure S3

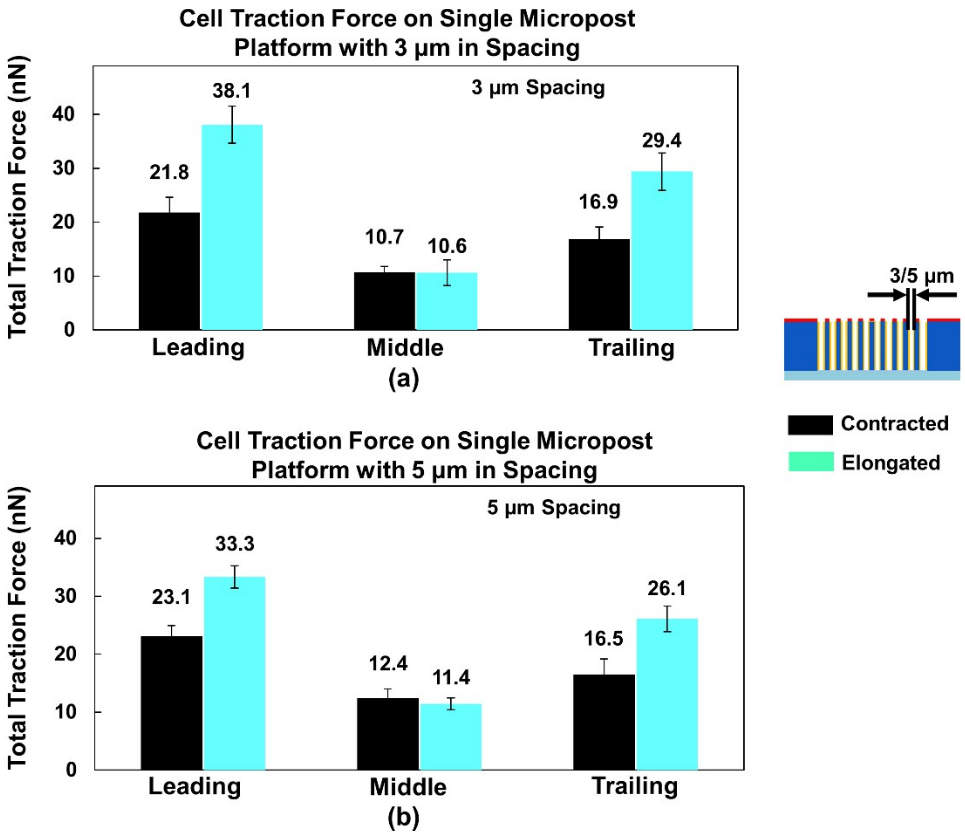

Figure S4
